# Dot-Matrix Hologram Rendering Algorithm and its Validation through Direct Laser Interference Patterning

**DOI:** 10.1038/s41598-018-32294-5

**Published:** 2018-09-24

**Authors:** Tomas Tamulevičius, Mindaugas Juodėnas, Tomas Klinavičius, Andrius Paulauskas, Kęstutis Jankauskas, Armantas Ostreika, Andrius Žutautas, Sigitas Tamulevičius

**Affiliations:** 1 Institute of Materials Science, Kaunas University of Technology, K. Baršausko St. 59, LT-51423 Kaunas, Lithuania; 2 Department of Physics, Kaunas University of Technology, Studentų St. 50, LT-51368 Kaunas, Lithuania; 3 Department of Multimedia Engineering, Kaunas University of Technology, Studentų St. 50, LT-51368 Kaunas, Lithuania

## Abstract

The fight against forgery of valuable items demands efficient and reasonably priced solutions. A security tag featuring holographic elements for anti-counterfeiting is one of them. However, the content and colours of a diffraction image that would be seen by an observer are often counterintuitive in the design stage. Here, we propose an original algorithm based on the conical diffraction formalism, which can be used to describe the variations of a diffraction image with respect to all aspects of observation. We validate the output of the algorithm by comparing it to test holograms, which we have produced by employing direct laser interference patterning (DLIP) in electrochemically grown nickel foil. We have employed a motorized femtosecond laser system to micro-machine arrays of 65 µm × 65 µm sized diffraction gratings with a defined orientation and pitch on the order of 1 µm. Based on completed diffraction efficiency measurements, we determined optimal ablation parameters, i.e. 57.4 mJ/cm^2^ fluence per pulse and 1100 pulses/pixel. Furthermore, we show how accurate the proposed algorithm is through measured diffraction spectra as well as captured diffraction images of test holograms produced using the obtained parameters. Finally, we showcase anti-counterfeiting tag prototypes with complex holographic effects, i.e. colour reconstruction, animation effects, and image multiplexing. The proposed algorithm can severely shorten the time between design and production of a holographic tag, especially when realizing it via a competitive origination technology—DLIP.

## Introduction

The level of product piracy and counterfeiting is increasing every day and demands innovative solutions to fight against it^1^. New anti-counterfeiting methods, utilizing physical unclonable functions^2^, light up-conversion modulation^3^, optical Moirés effects^4^, ripple formation^5^, holograms integrated into low-dimensional electronic devices^6^, metasurface holograms^7,8^, etc. are emerging, but the conventional holographic-lithography-based solutions are still leading in the market and their further growth is expected^9^. One of the most widely recognized hologram technology for anti-counterfeiting applications is the dot-matrix technique, where a design comprising of many tiny dots—which are too small to be seen by the naked eye (diameter usually bellow 100 µm) and each of which is a separate diffraction grating (a holo-pixel)—creates an impression of a 2D or a 3D image^9,10^. Historically, this technology used to employ continuous wave lasers of blue or ultraviolet wavelength and corresponding photosensitive materials^10–19^, but recent progress in ultrafast lasers has enabled a range of patterning capabilities on virtually any material without the need of photoresists. Direct Laser Interference Patterning (DLIP) technology, employing ultrashort laser pulses, was demonstrated on many different materials, including special inks^20^, polymers^1,21^, metals, ceramics, and coatings^22–24^. It was also scaled up via roll-to-roll printing^22,25,26^, but its practical applicability for anti-counterfeiting applications has been rather limited up to now^1,20,22^.

The main advantage of holography technology as an anti-counterfeiting method is the near-impossible difficulty of reproduction without the possession of specific equipment and knowledge. Moreover, vivid diffraction images are attractive and eye-catching, which adds to the visual appeal of the protected product. On the other hand, since the observed image depends on the illumination scene, design and validation of holograms is more complicated when compared to colour printing methods^27^. One of the ways to determine the authenticity of a hologram is by creating a database containing hologram images acquired under specific illumination/observation scenes. This can be achieved either by moving a camera and taking images under predefined angles (e.g. by using a robotic arm^28^ or a scanner^29^), or by fixing the camera and changing the illumination, e.g. by electrically switching a lamp array positioned on the surface of a hemisphere^30^. Once such database is available, validation of a hologram is possible through the use of automatic image matching combined with a special parametrization of an efficient, goal-oriented augmented reality user interface on a smart mobile—or any other—device that supports constrained navigation^28^.

Another approach for design and later validation of holograms—which we propose in this paper—can be implemented at the very first step of their production. Synthetic dot-matrix holograms and stereograms^31^ can be imposed by employing dedicated, physics-based algorithms, that take into account the laws of diffraction and help predefine the directionality of diffracted light^32^, kinetic effects, images, and animations as well as perceived colours and even human vision peculiarities. Positions of the light source, the observer, and the hologram itself; the spectral power distribution of the light source and the reflectance of the used material; the pitch, the orientation, the position, and the shape of each holo-pixel^12^; the luminosity of each graphic pixel^32,33^—all must be specified in order to achieve the effects intended by the graphic designer^27^. Additional degrees of freedom for proper hologram colour synthesis and equalization of angular divergence can be achieved via control of the duty cycle and the line curvature at the stage of hologram pattern design. However, these parameters are much easier to realize for holograms imposed by electron beam lithography^33^.

Application of DLIP for hologram origination presents a challenge to the well-established technologies, i.e. e-beam or conventional dot-matrix lithography, which besides being limited to typical substrates, costly, and time-consuming also require additional operations—like deposition of electron- or photo-resist and its development. In the frame of security tag realization for anti-counterfeiting applications, DLIP is a one-step microstructuring process and is an alternative to conventional lithography technologies. The end product (a hologram) can be imposed by a single ultrashort laser exposure step on a surface or even in bulk^34^ of practically any material. High peak power and energy density required by DLIP limits the applications of ultra-short pulse lasers equipped with optical setups based on the Spatial Light Modulator (SLM)^13,19^, however, adaptive optics and advanced diffractive optical elements (DOE’s) help to overcome this limitation and enable imposing security tags with a similar level of complexity^1,25^, which, in that case, depends entirely on its design.

A hologram diffraction image rendering algorithm, that could take into account a particular optical setup used for hologram origination, would enable to achieve the highest complexity and the best match to the design. Moreover, rigorous description of virtual hologram images for different observation/illumination scenes can be very helpful not only for preview of a hologram prior to lengthy fabrication process but also for evaluation of already manufactured security tag’s authenticity^27^. Use of such software tool was just recently announced by a reputable producer of holographic anti-counterfeiting solutions—Optaglio (Lochovice, Czech Republic)^35^.

In this work, we propose an algorithm based on the conical diffraction formalism, enabling the design and rendering of dot-matrix hologram diffraction images. We have employed and optimized a direct femtosecond laser interference lithography system—capable of controlling the pitch and orientation of interference fringes—for origination of dot-matrix holograms on a nickel foil. Through the produced test holograms, we then validate the algorithm by comparing its output to spatial and spectral diffraction properties observed under defined illumination scenes. We conclude the paper with a showcase of complete security tags with dot-matrix hologram effects, i.e. colour reconstruction and kinetic effects, and an original image multiplexing method, along with their rendered images.

## Results

**Figure 1:**
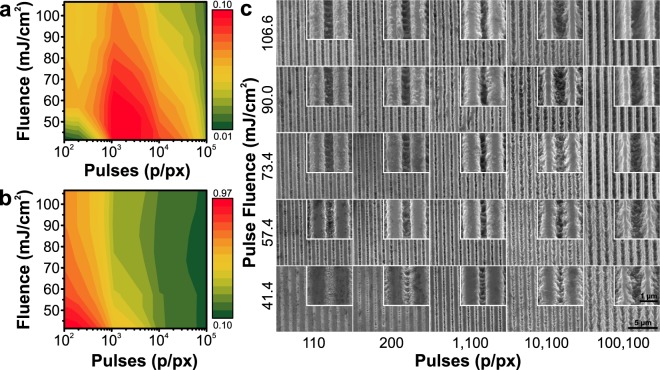
Optimization of the interference ablation process. First (**a**) and zero (**b**) order diffraction efficiencies measured from the test holograms with varying number of applied pulses (110–100100 pulses per holo-pixel) and varying the pulse fluence (41.4–106.6 mJ/cm^2^). (**c**) A set of SEM micrographs of the imposed grating structures at two characteristic magnifications.

The smallest indivisible part of a dot-matrix hologram is a diffraction grating. Diffraction efficiency of gratings strongly correlates with their quality^26^ which depends on applied laser processing conditions^36,37^. Therefore, first of all, we performed an optimization of these parameters by varying the fluence (41.4–106.6 mJ/cm^2^, calculated after measuring the laser power incident on the sample) and the number of applied pulses per pixel (110–100100 p/px, defined by *P* = 100 + 10^*n*^, where *n* is the column number) to achieve the highest relative diffraction efficiency (here and throughout the paper, the diffraction efficiency is a ratio of diffracted light power relative to a reflection off a pristine Ni surface). An array of test dot-matrix holograms (50 by 50 pixels) was recorded for diffraction efficiency analysis. Dependence of the zero and first order diffraction efficiency on the laser processing parameters together with the resulting grating micrographs are summarized in Fig. 1. We selected 57.4 mJ/cm^2^ fluence and 1100 p/px as the favourable conditions to obtain the highest diffraction efficiencies in this particular case.

**Figure 2:**
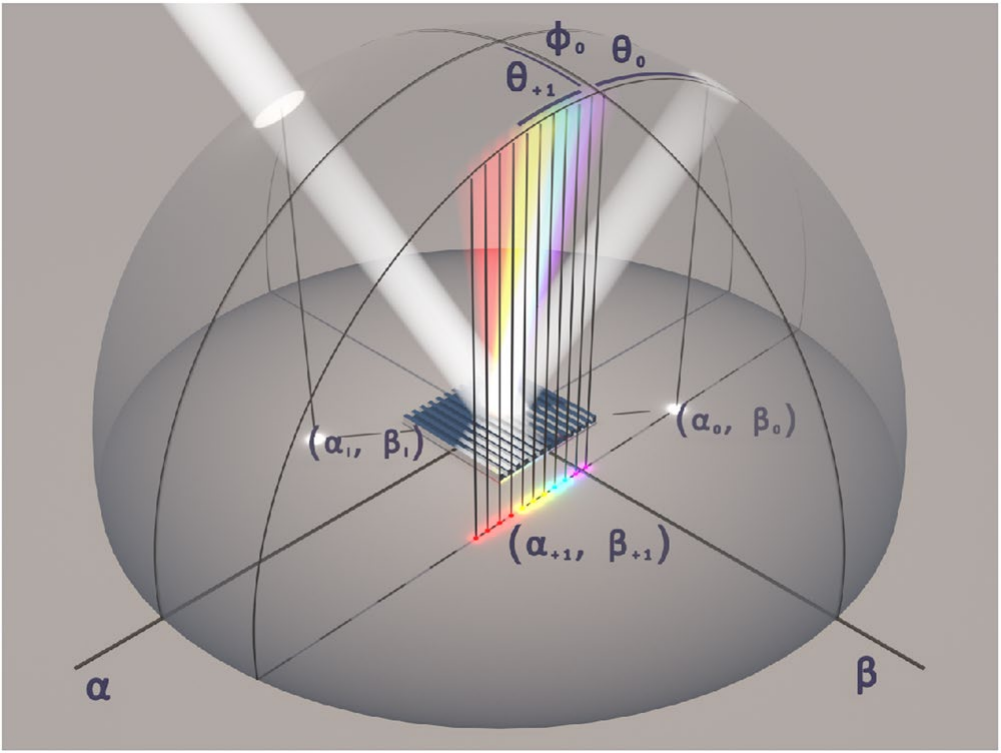
Conical diffraction. Positions of incident light and diffraction orders produced by a reflection grating in real space (hemisphere). Description in terms of angular coordinates (θ, ϕ) and direction cosine space (α, β plane) is shown (“i” – incident, “0” – specular reflection, “+1” – first diffracting order for an arbitrary oblique incidence). After^38^.

**Figure 3:**
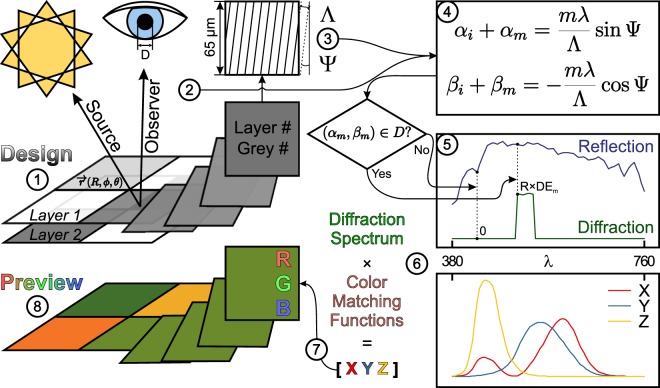
Hologram rendering algorithm. The conversion path of a single pixel from a digital image into a rendered hologram is showcased. “1” Design of a hologram, representing different pitches and orientations in layers of grey scale images; “2” Setup of an observation scene ($\vec{r}$ – position vector in spherical coordinates (R, φ, θ), D – aperture diameter); “3” Conversion of input data into pitches (Λ) and orientations (Ψ) of holo-pixels; “4” Application of the conical diffraction formalism^38^ (notations are explained in the supplementary material online); “5” Construction of the diffraction spectra visible through the defined aperture (taking into account the reflectance (R) of the material and diffraction efficiency (DE) of the grating); “6” Spectrum conversion into tristimulus values in the 1931 CIE XYZ colour space (colour matching functions were taken from tables in^39^); “7” Conversion of the colour of a holo-pixel into RGB scale; “8” Preview of a rendered hologram image.

We have prepared an original hologram rendering algorithm based on the conical diffraction formalism, initially described by^38^ (see Fig. 2 and a theoretical consideration in the Supplementary Information online), in MATLAB (Natick, MA, USA, MathWorks). The algorithm can be divided into eight steps as depicted in Fig. 3. The hologram design files must be 8-bit grey scale bitmaps of the PNG file format. A separate file—that is treated as a layer—is required for every grating period that is supposed to be different (Fig. 3“1”). The available grating pitches are hard-coded in the algorithm and mapped to corresponding layer numbers, whereas the grating orientation is up to the designer to decide via the grey scale values. The software reads the files, detects the layer count and the number of grey scale levels within each layer, and suggests parameters for holo-pixels, i.e. a pitch for each layer and a grating orientation for each grey scale level within each layer (Fig. 3“3”). The illumination and observation scenes as well as the observer’s aperture size are selected prior to the calculation of the hologram image (Fig. 3“2”). Each pixel of the provided bitmap images is treated as a single 65 µm × 65 µm holo-pixel. The algorithm scans through the visible range of wavelengths for each holo-pixel, applies the conical diffraction formalism for the observation setup (Fig. 3“4”), and finds a spectrum of diffracted light that can pass through the defined aperture at the selected observer position (Figs 2 and 3“5”). These spectra are converted first into 1931 CIE XYZ colour space through eq. (1)^39^ (Fig. 3“6”) and then into RGB values via eq. (2)^40^ (Fig. 3“7”). These values are used to render and display the final hologram image (Fig. 3“8”).

1 $${[XYZ]}_{out}=\frac{{\sum }_{\lambda }[XYZ(\lambda )]\cdot D65(\lambda )\cdot R(\lambda )\cdot D(\lambda )}{{\sum }_{\lambda }XY{Z}^{Y}(\lambda )\cdot D65(\lambda )},$$

where [*XYZ*]_*out*_ – tristimulus value vector, *XYZ*(λ) – a colour matching function for the standard CIE 1931 observer, *D*65(λ) – spectral intensity of the D65 standard illuminant, *R*(λ) – reflectivity (taken from tables in^41^), *D*(λ) – diffraction efficiency.

2 $$[RGB]=[K]\cdot [XYZ],$$

where [*RGB*] – a colour vector, [*K*] – a matrix for conversion from XYZ tristimulus values to RGB, [*XYZ*] – vector of CIE 1931 tristimulus values.

**Figure 4:**
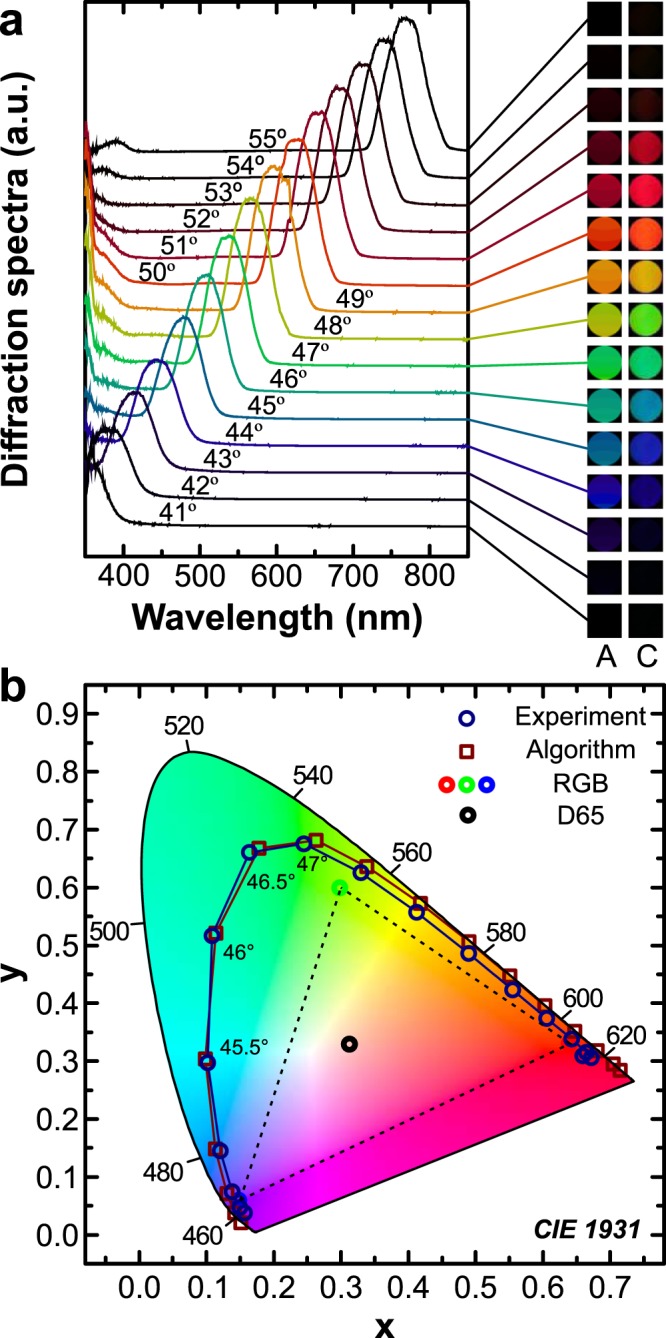
Test hologram analysis. (**a**) Spectral composition of the diffraction spectra and hologram images rendered by the algorithm (a, “A”) next to camera snapshots (**a**, “C”) obtained from a single pitch (Λ_3_ = 1.016 µm) test hologram under different angular positions. Angle values indicated on the spectra are the angles of incidence θ_i_. (**b**) Colours of the hologram obtained from the algorithm (squares) and from the acquired spectra (circles) on a CIE 1931 diagram. The standard RGB (sRGB) triangle along with a standard illuminant D65 are also plotted for reference.

We validate this algorithm through test holograms, which we have produced by employing the direct laser interference patterning setup operating in the optimized conditions. Analysis of the test holograms provided three kinds of results: (i) diffraction spectra containing information about the colour of the diffracted light (Fig. 4(a)), (ii) camera images showing the actual hologram (Fig. 4(a)“C”), and (iii) hologram images rendered using the algorithm (Fig. 4(a), “A”). We analysed the test diffraction gratings in the classical configuration where the grating grooves are perpendicular to the plane of incidence (see Materials and methods for the description of the analysis of diffraction images). To investigate further, we devised a hologram consisting of four different pitch but equal size areas. The results of this experiment are summarized in Supplementary Fig. S1.

To further demonstrate the capabilities of the algorithm and the used DLIP system, we have recorded more complex holograms that could represent the prototype of the end-product. In the following examples we demonstrate three types of hologram effects: colour reconstruction, animated effects, and image multiplexing.

**Figure 5:**
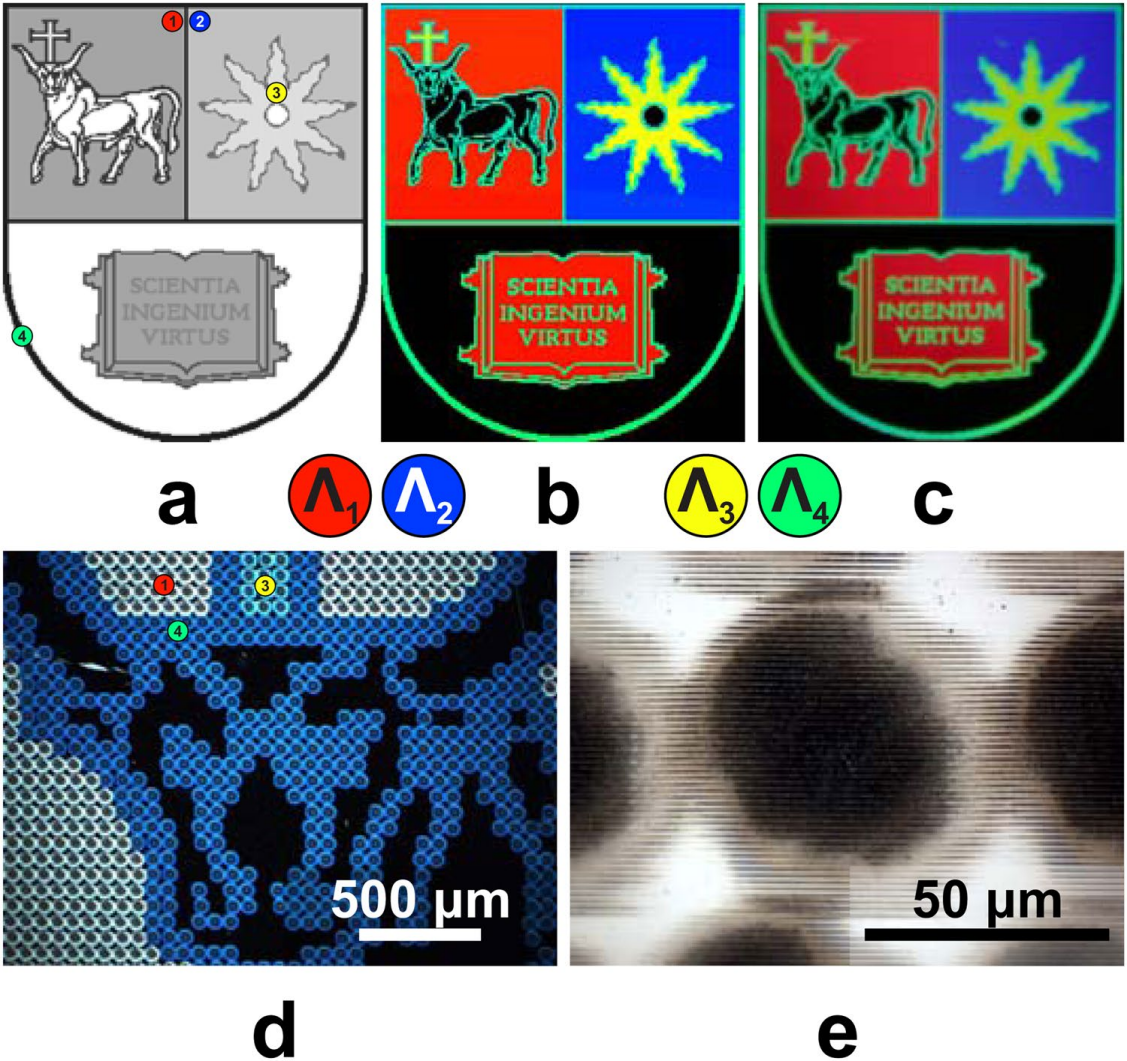
Colour reconstruction. (**a**) The design, (**b**) rendered hologram image, and (**c**) camera image of the coat of arms (example of structural colouring, size 249 by 300 px); (**d**) dark-field optical microscope micrograph with three different pitch grating zones, (**e**) bright field image of a holo-pixel.

The colour reconstruction is demonstrated on the coat of arms of Kaunas University of Technology (Fig. 5). Here, red, blue, and yellow colours were ascribed to corresponding gratings that produce them under a 30° angle of illumination (see Supplementary Fig. S2). We chose the contour to be green as the black colour could only be achieved by an intact surface, which had already been reserved by a part of the coat of arms, i.e. the background. Figure 5 depicts the merged layers of the hologram design with five uniform colour areas representing different grating pitches (Fig. 5(a)), the rendered hologram image (Fig. 5(b)), and a camera snapshot of the hologram (Fig. 5(c)). Dark-field illumination enables direct visualization of the different pitch holo-pixels in colour (Fig. 5(d)). A closer look of a single dot matrix holo-pixel is depicted in (Fig. 5(e)).

**Figure 6:**
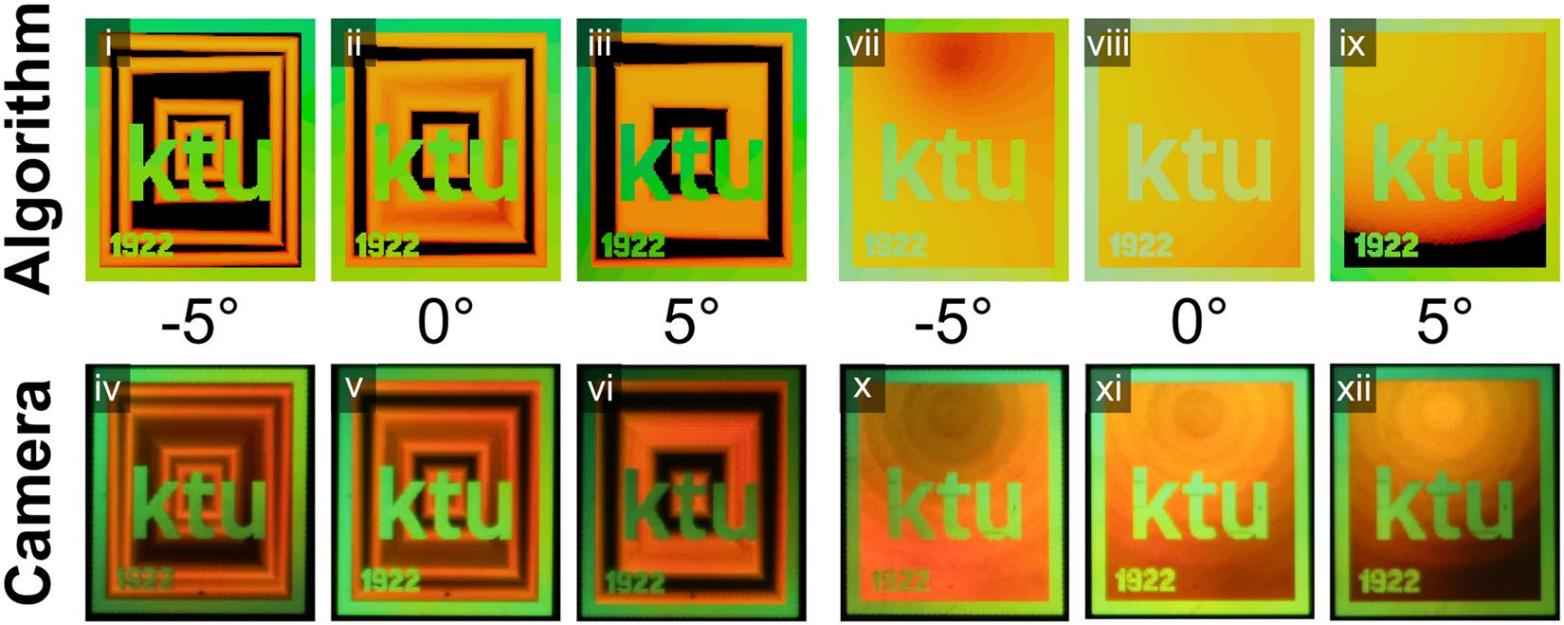
Kinetic effects. The logo of Kaunas University of Technology (examples of the hologram design, size 131 by 150 px, animated images obtained at continuous angle rotation are presented in Supplementary Animation S3 and S4).

Typical kinetic hologram animation effects, like zoom-in-zoom-out^12^, changing shape^12^, etc. can be inscribed by gradually changing the orientation of the gratings. We have modified the background of the logo of Kaunas University of Technology to contain two types of typical effects used in the dot-matrix holograms: zooming squares (Fig. 6(i–vi), Supplementary Fig. S3) and zooming sphere (Fig. 6(vii–xii), Supplementary Fig. S4). The acquired and rendered images are achieved by rotating the sample around its normal.

**Figure 7:**
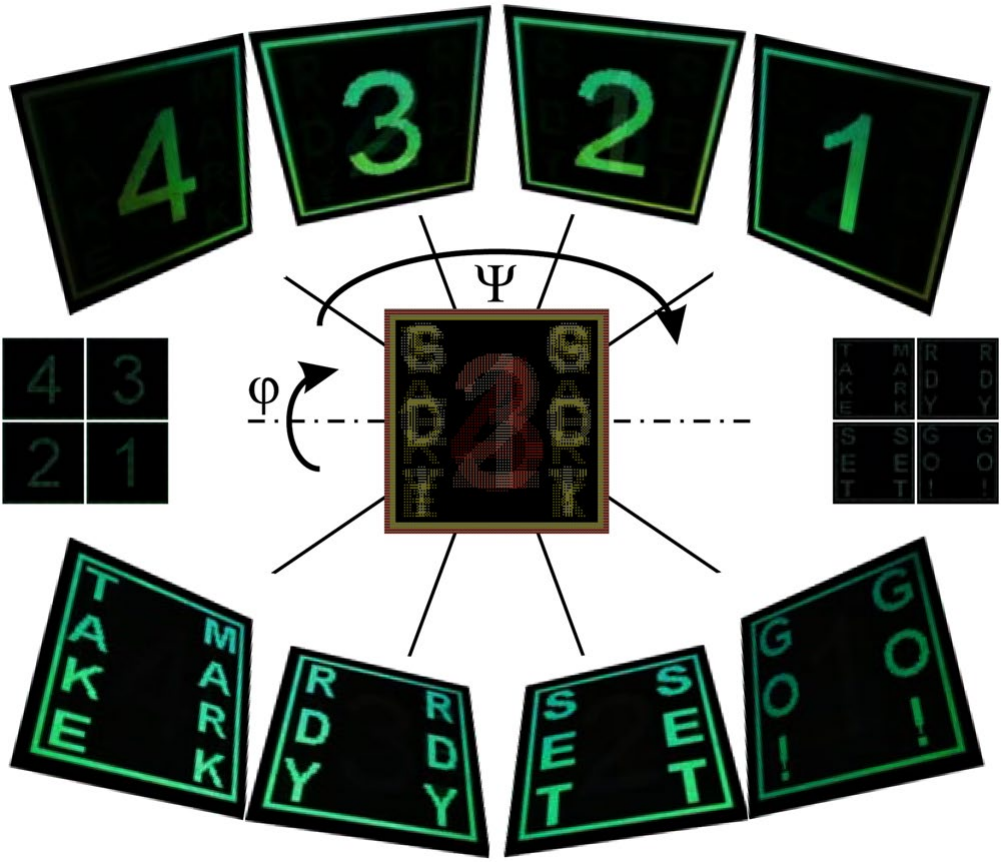
Multiplexing example. Countdown sequence (example of two possible multiplexing schemes: Ψ rotation for numbers 4–1 and text as well as switching between the numbers and the text through the sample rotation (φ); 160 by 160 px, animated images obtained at continuous angle rotation are presented in Supplementary Animation S5)).

A wide range of available pitches in the DLIP system (showcased in Supplementary Fig. S2) opens the opportunity for an additional degree of freedom in image multiplexing as demonstrated in the final example. Changing the grating orientation in adequate steps as well as its pitch enabled us to spatially separate images seen by an observer at a fixed position, thus achieving an original image multiplexing technique. In Fig. 7, multiplexing of a 4 to 1 countdown sequence is depicted. We demonstrate that one can separate images using two different periods with an adequate difference in pitch. After selecting the pitch for the first image, one can find the second pitch by changing the observation scene until visible light no longer falls into the observer’s aperture and checking which pitch starts to diffract visible light into it. Thus, images can be switched not only by changing the Ψ angle (or rotating in the αβ plane, see Fig. 2), but also by rotating around a horizontal axis (in turn changing the angle of incidence and the angle of observation accordingly). Under 36° sample rotation, text “TAKE MARK”, “RDY”, “SET”, “GO!” appears under the same Ψ angles as the countdown sequence appeared under a 30° sample rotation. The rendered diffraction images under continuous Ψ and φ rotation are depicted in Supplementary Fig. S5.

## Discussion

The diffraction efficiency measurements were performed with a sole purpose of selecting the optimal DLIP processing conditions (Fig. 1) in order to achieve the maximum diffraction efficiency and the most vivid image of the produced anti-counterfeiting tag. However, when comparing the first (Fig. 1(a)) and zero (Fig. 1(b)) order diffraction efficiency dependencies on the laser processing parameters, one could expect to see that the intensities follow π shifted harmonic dependencies on phase shift that is related to the structure height and wavelength, like it is depicted in Supplementary Fig. S6(b) and described in^42–44^. Firstly, it should be noted, that both DLIP processing parameters, the fluence and the number of pulses, have influence on both the groove depth and the filling factor (which is a ratio of grating ridge width to its pitch), thus a direct comparison between the experimental Fig. 1(a,b) and simulated Supplementary Fig. S6(a) is inappropriate. Nevertheless, such dependence is only valid for a certain range of filling factor (see Supplementary Fig. S6(a)) and experimental studies (SEM and AFM measurements) show that the produced filling factor falls out of this range (we estimated a filling factor of 75% achieved by using the optimal processing conditions). As can be seen in the last two columns of Fig. 1(c), where a high number of pulses was applied, both first and zero order diffraction efficiencies decreased (see Fig. 1(a,b)). The surface must have become less reflective, mostly because of the deterioration of groove surface quality. In the case of lower number of applied pulses, the laser had ablated very little material, but it still changed the surface reflectivity, so the structures acted more like an amplitude grating. In addition, the presence of ablation debris (unavoidable due to the sheer number of applied pulses) on the grating ridges might have some influence on the diffraction efficiency.

Based on measurements of the filling factor in the SEM images, AFM profiles, as well as experimental diffraction efficiency measurements (Fig. 1), together with numerical simulation results (see Supplementary Fig. S6), i.e. by employing the scatterometry^45,46^ technique, the depth of the structures imposed at optimized conditions (57.4 mJ/cm^2^ and 1100 p/px) can be estimated to be approx. 210 nm. This prediction was confirmed to some extent by AFM measurements, which showed a 150 nm average of minimum groove depth (see Supplementary Fig. S7). The mismatch between the predicted and measured depth can be related to the fact that the holo-pixels were produced using a Gaussian intensity distribution, meaning that the groove depth varies within the holo-pixel (see Supplementary Fig. S8) as well as scatterometry technique being a strictly averaging measurement of several holo-pixels, whereas AFM measurement took into consideration only a ¼ of the holo-pixel area. One can expect that more material could be removed by increasing the fluence. However, at a certain level the interference minimum passes the ablation threshold and the grooves of the grating can no longer get deeper—the whole holo-pixel is getting deeper instead (see Supplementary Fig. S8). This phenomenon was already described in DLIP experiments of polymer materials in^1,21^. On the other hand, as seen in the numerical simulation results depicted in Supplementary Fig. S6, any further increase in diffraction grating groove depth would not increase the diffraction efficiency at the given filling factor, however, a filling factor closer to 50% should be achieved in order to reach the theoretical 46% efficiency. From the practical point of view, since a manufactured hologram usually contains many pixels, the necessary amount of pulses is the limiting speed factor, therefore a higher fluence and a smaller number of pulses are preferable.

Other authors report similar fluence values for ablation of nickel. 0.36 J/cm^2^ was used for DLIP with a laser of 1064 nm wavelength and 10 ps pulse duration^47^. Using longer pulses of 6 ns and 1053 nm wavelength, 2.2 J/cm^2^ fluence was selected by^48^. It was also demonstrated that using a reasonable pulse overlap one can make the grating continuous—557 mJ/cm^2^ fluence and 95% overlap led to deepest structures in^26^. Ablation of invar (36% nickel Fe-Ni alloy) with a focused femtosecond laser of 1060 nm wavelength and 350 fs pulse duration was also reported. The ablation threshold was found to be 0.122 J/cm^2^ and the best structure quality was obtained using 389 pulses and peak fluence of 0.55 J/cm^2^, reaching diffraction efficiencies of 9%^36^. These values are close to the ones presented in Fig. 1, however, in our case, the used pulse fluence was lower. Compared to these reports, the difference can be attributed to three main causes: a) percussion ablation mode as opposed to continuous scanning of the interference pattern (continuous scanning, even at a high apparent pulse overlap, such as 95% discussed above, still results in just 20 pulses per area, which is significantly less than what we use), b) 515 nm used wavelength, where nickel has lower reflectivity than in the infrared, and c) shorter pulse duration of approx. 300 fs. It should be pointed out that the presented results add to the research presented by other authors and continue to prove DLIP as a rapid patterning technique which is compatible with materials conventionally used in production of anti-counterfeiting security tags, i.e. nickel foil and plastics used for thermal embossing^26,48^.

The optimization of DLIP allowed us to produce efficient holograms for validation of the proposed holographic image rendering algorithm. A couple of points here are also worth discussing. One can see that the diffraction spectra follow the Bragg’s diffraction law (see Supplementary Eq. (S1)) as expected—the intensity peaks are linearly shifted with respect to the angle (see Supplementary Fig. S1(c)). At certain angles of incidence, there is a spatial overlap of visible light between the end of the first and the beginning of the second diffraction orders, i.e. the acquired spectra spanned over the wavelengths of the free spectral range. That is easily distinguishable in the measured spectra as secondary peaks of much lower intensity (see a peak around 400 nm in Fig. 4(a)). For the given observation scene this effect is much more prominent using gratings of different pitch (see Supplementary Fig. S1(b), where a case with several gratings is presented). This spatial overlap, however, adds an additional point to consider. Even though the secondary peak is significantly less intense, it still contributes to the final perceived colour. This can lead to unintentional colour mixing (red and blue would produce the magenta colour, otherwise unavailable in the colour spectrum). One can see this colour mixing in the comparison of camera images and algorithm’s output in Fig. 4 and Supplementary Fig. S1.

That being said, a close overall match can be observed between the angular spectral measurements, the camera images, and the rendered hologram images. The colours in CIE xyY coordinates extracted from the diffraction spectra are very close to the ones predicted by the suggested hologram image algorithm (Fig. 4(b)). Slight discrepancy between the images rendered by the algorithm (Fig. 4(a)“A”) and camera images (Fig. 4(a)“C”) can be attributed to inaccurate representation of arbitrary colours in RGB scale (notice the distance between the experimental points and the sRGB triangle), colour pre-processing by the camera hardware, and camera positioning errors.

The range of colours covered and their distance from the CIE outer curve displays how close these holograms are to the true-colour spectrum. Better results might be obtained by carefully selecting the illumination source as described in^12^. By mixing particular colour channels and tuning their intensities, even white light can be obtained^33,49^. It should be noted, that the colour purity is directly related to the observation distance and aperture size (which in our case was 60 cm and 72 mm respectively), as the further the observer’s position, the smaller the range of the wavelengths that get through an aperture, resulting in narrower diffraction intensity peaks, i.e. higher perceived colour purity.

The presented hologram examples (Figs 5, 6, 7, S3, S4, S5) exhibit the complexity that can be achieved by merging the capabilities of a femtosecond DLIP system and the proposed hologram image rendering algorithm. However, not all of the available diffraction grating pitches can be used for construction of a single hologram image due to the spatial distribution of the diffraction orders produced by different gratings. On the other hand, this creates a potential for an additional level of multiplexing complexity. Not only can images be separated through the orientation of holo-pixels, but also through the pitches of the gratings. Supplementary Fig. S2 summarizes the hologram colours that can be observed in a typical scene provided by the 15 grating pitches available in our system. It is assumed that the observer is looking at the hologram at a normal angle and the light is illuminating the hologram at 30° and 45° angles of incidence. The selected range of angles used to describe colours was also reported in^12,32,49^ and represents typical illumination conditions in real life applications. The range of typical pitches—that in the case of classical grating configuration reconstruct visible colours—usually spans 940–1254 nm for the angle of incidence of 30°^12^ and 667–944 nm for 41–45°^32,49^. Similar image multiplexing performance was demonstrated on chiral metasurface structures^8^, dot-matrix holograms in photoresist^10,32^, electron resist^33^.

It is evident, that the algorithm proposed in this work is able to accurately render sets of diffraction images seen at determined illumination scenes and enables better visual perception of the effects inscribed in the dot-matrix hologram. The accuracy of the algorithm enabled the design of complex holograms without the need of a slow iteration process—involving the manufacture of useless holograms that in the end do not produce intended effects—and also evaluation of the authenticity of existing elements^27^. The validation examples demonstrated that the conical diffraction formalism applied in the original algorithm enables precise reconstruction of images produced by arbitrarily oriented gratings, which faces major limitation when only classical diffraction equations are considered. Furthermore, design of kinetic effects using the algorithm becomes straightforward and does not require full knowledge of the conical diffraction, meaning that an unqualified user can see the final product and can fine-tune it before manufacturing.

## Conclusions

It was demonstrated that femtosecond laser and diffractive optical element based two beam *4 f* interference lithography setup can be used for patterning of sub-micron pitch diffraction grating in electrochemically grown nickel foil. By varying the fluence per pulse and the number of pulses it was shown that relative diffraction efficiency is highest at 57.4 mJ/cm^2^ fluence and 1100 p/px and reaches 10%. AFM measurements revealed that in this case the structure height is around 150 nm. An algorithm based on the conical diffraction formalism for rendering of hologram diffraction images was created and validated by employing origination of holograms via DLIP and their experimental characterization. It was demonstrated that current optical femtosecond laser based DLIP setup together with the proposed algorithm is capable to originate dot-matrix holograms with predefined colours, animation effects, and multiplexed images that can be applied in production of anti-counterfeiting tags.

## Materials and Methods

### Production of gratings

Linear diffraction gratings in the range of micron and sub-micron pitch were imposed in a nickel film, which was deposited by employing an electroplating technique on a flat, electrically conductive substrate. Thickness of the film was 50 µm on average. More details about the experimental conditions used in electroplating process for growth of the nickel foil can be found in^17^.

The direct two laser beam interference ablation process was performed by employing the second harmonic (515 nm) of Yb:KGW femtosecond laser Pharos (Vilnius, Lithuania, Light Conversion). The laser pulse repetition rate was fixed at 40 kHz. The laser pulse picker, optical intensity attenuation, the control of interfering laser beams, and sample translation—all were implemented by employing a FemtoLab (Vilnius, Lithuania, Altechna R&D) laser workstation. More detailed information about the setup can be found in^50,51^. Dot-matrix hologram images were imposed by positioning the sample using XYZ translation stages (Pittsburgh, PA, USA, Aerotech) controlled via SCA software (Vilnius, Lithuania, Altechna R&D). Circular polarization was used in the experiments. Further description can be found in the Supplementary information.

The dot-matrix hologram images were imposed by selecting a necessary number and controlling the energy of laser pulses per single holo-pixel and by translating the sample between the neighbouring pixels. Depending on the task performed, either command line with point coordinates and grating orientations, or corresponding resolution bitmap images were used as input for the laser control program.

### Analysis of diffraction efficiency

Diffraction gratings of the dot matrix holograms were investigated using a scanning electron microscope (SEM) Quanta 200 FEG (Hillsboro, OR, USA, FEI), an atomic force microscope (AFM) JPK NanoWizard 3 (Berlin, Germany, JPK) operated in AC mode employing ACTA probe (Mountain View, CA, USA AppNano), and a BXFM microscope (Tokyo, Japan, Olympus) with bright-field and dark-field illumination, MPlanFLN 5 × 0.15BD FN26.5 and MPlanFLN 100 × 0.90BD FN26.5 objectives, and digital 3.3 MP Charge-Coupled Device (CCD) colour camera MP3.3-RTV-CLR-10 (Surrey, BC, Canada, QImaging). Relative diffraction efficiency of the test hologram was evaluated by measuring the intensity of the diffracted light emitted from a 650 nm wavelength laser diode and normalizing it to the reflectance off pristine Ni surface. The intensity of the diffraction spectrum was measured with a PD300UV photodiode sensor and Nova II power meter (Jerusalem, Israel, Ophir).

Diffraction efficiency simulations of an idealized rectangular shape microstructures were performed by employing Rigorous Coupled Wave Analysis (RCWA) method-based software GSolver (Allen, Texas, USA, Grating Solver Development Company). The models were calculated for a perpendicular incidence of TE polarized, 650 nm wavelength light on a 1016 nm pitch diffraction grating in nickel. The groove depth of the structure was varied from 0 to 1000 nm with a step of 1 nm and the structure’s filling factor from 0 to 100% with a step of 1%.

### Analysis of diffraction images

**Figure 8:**
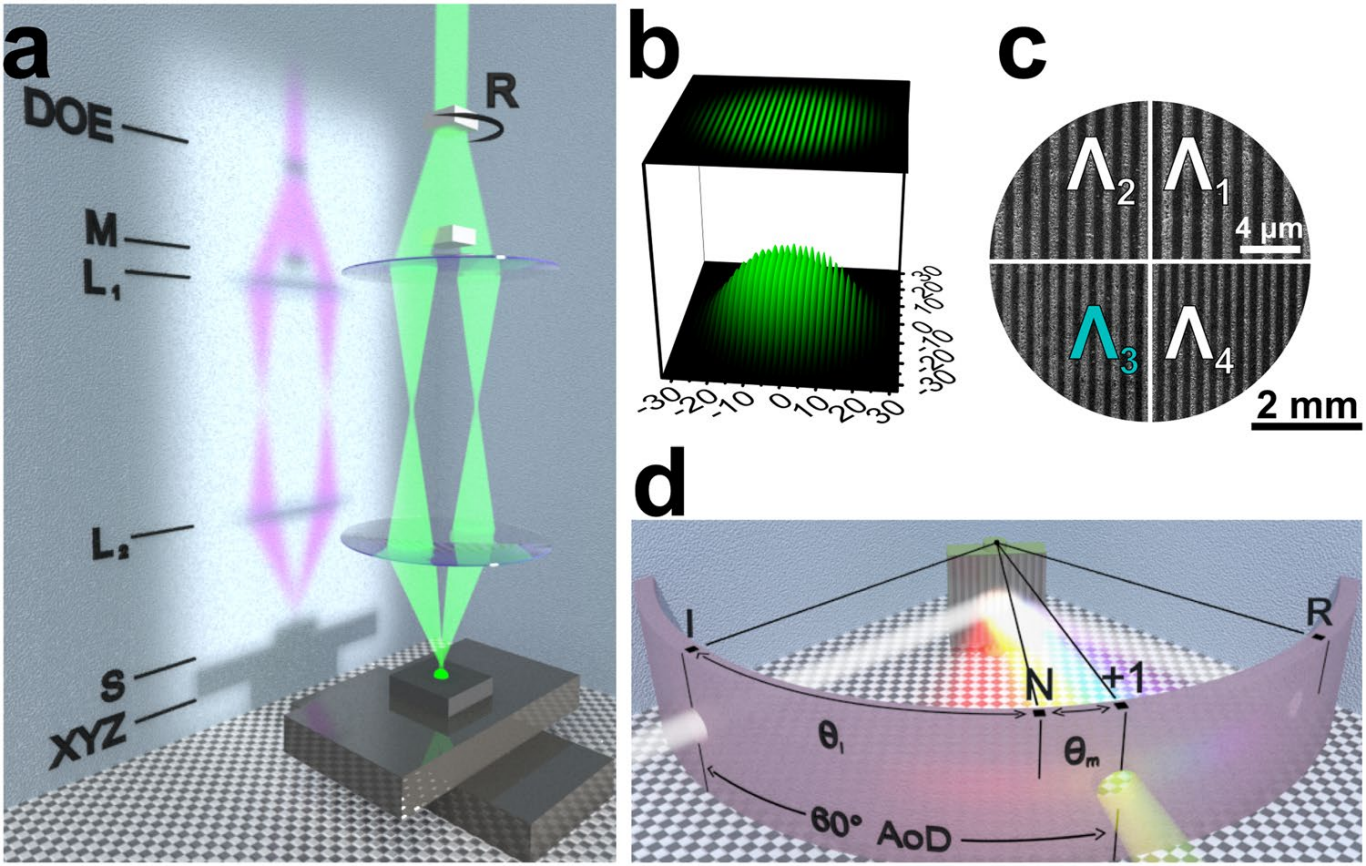
Experimental methods. (**a**) Principle scheme of the symmetrical 4 f two laser beam interference ablation setup. From the top: a set of diffractive optical elements (“DOE”) on a rotational stage (“R”); a mask for blocking the zero and higher diffraction orders (“M”); two lenses (“L1”, “L2”); the sample (“S”); translation stages (“XYZ”). (**b**) Simulated interference fringes of two beams with Gaussian intensity distribution. (**c**) Schematics of a dot-matrix hologram with four different grating pitches (Λ_1-4_) used for validation of the rendering algorithm. Scanning electron microscope images of the actual gratings obtained under the same magnification are depicted in the respective quadrants. (**d**) Principle scheme of the setup used for spectral and spatial investigation of the diffracted light including the angle notation and experimental conditions used for spectral and image acquisition (“I” incident light, “R” specular reflection, “N” grating plane normal, “+1” first diffraction order, θ_i_ and θ_m_ are the angles of incidence and diffraction, “AoD” denotes the constant angle of detection).

Simplified test hologram images, containing either one or four identical in size grating areas (see Fig. 8(c)), were analysed by combining three methods: (i) measurement of the diffraction spectra of the produced grating, (ii) capturing of images using a digital single-lens reflex camera (DSLR), and (iii) rendering of diffraction images through the proposed algorithm. For methods (i) and (ii), an automated custom-built goniometric setup was employed. A collimated white light beam from an incandescent light bulb was impinging on the sample. Sample rotation and the angle of spectra acquisition (detector position) were controlled using a custom software written in LabVIEW (Austin, TX National Instruments Corporation). More detailed information about the used setup can be found in^52^. Diffraction spectra were collected with a 6 mm diameter lens attached at the end of an optical fibre, which was connected to a 1.2 nm resolution optical spectrometer AvaSpec-2048 (Apeldoorn, The Netherlands, Avantes), operating in 350–890 nm wavelength range. Both the spectra and the hologram images were acquired by rotating the sample so that the angle of incidence varied (40° ≤ *θ*_i_ ≤ 60°) with a step of 0.5°, while keeping the angle of detection constant at 60° (see Fig. 8(d)). The acquired diffraction efficiency spectra were converted to 1931 CIE XYZ colour space using eq. (1) for comparison with the algorithm output (in the more complicated case of several different gratings, the spectra were also fitted using Normal distribution functions and separated from each other into individual datasets (see Supplementary Fig. S1(d)).

Meanwhile, images of the hologram at the same angle of detection were taken with a digital camera fixed on a tripod (method (ii)). DSLR camera 700D (Tokyo, Japan, Canon) and EF-S 18-200 mm f/3.5-5.6 IS (Tokyo, Japan, Canon) objective with a 72 mm diameter aperture were used. Distances from the sample to the fibre collecting lens and from the sample to the camera objective lens were selected in a way to maintain a similar solid angle of light collection.

## Electronic supplementary material

Supplementary information

Supplementary Figure S3

Supplementary Figure S4

Supplementary Figure S5

## Data Availability

All of the data that support the findings of this study and is not included in the supplementary information is available from the corresponding author upon reasonable request.
