# Supplementary material for: Dot-Matrix Hologram Rendering Algorithm and its Validation through Direct Laser Interference Patterning
Source: Sci Rep. 2018 Sep 24;8:14245. doi: 10.1038/s41598-018-32294-5 (PMC6155248; doi:10.1038/s41598-018-32294-5)
Supplement: Supplementary file 1 — Supplementary information [file 41598_2018_32294_MOESM1_ESM.docx]

**Dot-Matrix Hologram Rendering Algorithm and its Validation through Direct Laser Interference Patterning**

Tomas Tamulevičius, Mindaugas Juodėnas, Tomas Klinavičius, Andrius Paulauskas, Kęstutis Jankauskas,
Armantas Ostreika, Andrius Žutautas, Sigitas Tamulevičius

**Supplementary Information**

**Contents:**

1. Theoretical consideration
2. Direct laser interference patterning setup
3. Supplementary Figures S1-S8
4. References

**Theoretical consideration**

In a classical configuration, when the plane of incidence, i.e. the plane containing the incident beam and the normal to the grating surface, is perpendicular to the lines/grooves of the diffraction grating, the diffraction of light can be described by the grating equation ^1^:

$m\lambda=\Lambda\left(\sin\theta_{i}+\sin\theta_{m}\right)$, (S1)

where *m* – the order of the diffracted wave (*m* = 0, ±1, ±2, ±3, …), λ – the wavelength of light, Λ – the pitch of the grating, θ_i_ – the angle of incidence, θ_m_ – the angle of diffraction of *m*^th^ order.

However, a dot-matrix hologram usually consists of multiple pixels containing arbitrarily oriented gratings. Moreover, they may be illuminated at arbitrary oblique angles when observed in natural conditions. In such a case, the diffraction orders no longer lie in the same plane as the incident and reflected light like eq. (S1) predicts, instead they follow the surface of a cone, whose base is a plane cutting a hemisphere (see Fig. 2) and perpendicular to the grating grooves. In ^1^ it was explained how such a situation can be described analytically in terms of the direction cosines of propagation vectors. Using the proposed formalism, α_i_ and β_i_ are denoted as the direction cosines of the incident beam, α_0_ and β_0_ are the direction cosines of the undiffracted, i.e. specularly reflected, beam, whereas α_m_ and β_m_ are the direction cosines of the diffracted beam of the *m*^th^ order (see Fig. 2). The angular coordinates in Cartesian space are θ and φ, where φ is the angle in the plane parallel to grating grooves and their normal, and θ is the subsequent rotation in a plane perpendicular to the grating grooves (i.e. in the cone base plane) as shown in Fig. 2. The subscripts are the same as those associated with α and β ^2^. , and are direction cosines of the position vector $\vec{r}=\left(x,y,z\right)$ of the observation point in the Cartesian coordinates. The direction cosines are additionally related in a way so that $\alpha^{2}+\beta^{2}+\gamma^{2}=1$, indicating that only real, propagating diffraction orders are included and all non-propagating, evanescent orders, that would provide sum higher than one, can be neglected. Using this nomenclature, the incident and diffracted light can be described ^1,3^:

$\begin{matrix} \alpha_{i}=-\sin\theta_{0}\cos\varphi_{0}\text{; }\alpha_{m}=\sin\theta_{m}\cos\varphi_{0} \\ \beta_{i}=-\sin\varphi_{0}\text{; }\beta_{m}=\sin\varphi_{0}\text{ } \end{matrix}$ (S2)

Then the general grating equation for arbitrarily oriented diffraction grating lines is given by ^1^:

$\begin{matrix} \alpha_{m}+\alpha_{i}=\frac{m\lambda}{\Lambda}\sin\Psi\\ \beta_{m}+\beta_{i}=-\frac{m\lambda}{\Lambda}\cos\Psi\end{matrix}$, (S3)

where Ψ is the angle between the direction of the diffraction grating lines and the *α* axis (Ψ = 90° in Fig. 2). It should be noted that this formalism was also applied for extreme UV light beam splitters ^3^ and off-plane grating spectroscopy ^2^.

**Direct laser interference patterning setup**

The laser beam was diffracted into two first order diffraction beams of equal intensity by employing a set of DOEs mounted on computer-controlled stages (Fig. 8 (a)). The custom-made DOE element, tailored for the used laser wavelength, consists of a set of different pitch linear diffraction gratings, whose depth was selected to ensure a necessary phase shift ^4-6^. In order to have a high holo-pixel density and aiming at a more homogeneous intensity distribution, a square shaped laser beam profile was formed by cropping a Gaussian beam that was expanded and passed through a square aperture prior to entering the DOE. Residual intensity of the zero and higher order (*m* > |±1|) diffracted beams were blocked with a mask. A 4*f* lens system ^7,8^ was used to make the diffracted beams parallel and finally overlap them on the sample surface (Fig. 8 (a)). A simulated interference pattern of two Gaussian intensity profile laser beams, based on the calculations presented in ^9^, is depicted in Fig. 8 (b). Orientation of the interference fringes was controlled by rotating the DOE, while the spacing of the pattern was varied by changing the DOE to the one with a necessary grating pitch. The pitch of the imposed grating (Fig. 8 (c)) depends on the angle between the two interfering beams (which depends on the DOE chosen):

$\Lambda=\lambda/2\sin\left(\Theta\right)$, (S4)

where λ is the wavelength of the laser beam and Θ is the half angle between the two incident beams ^8^.


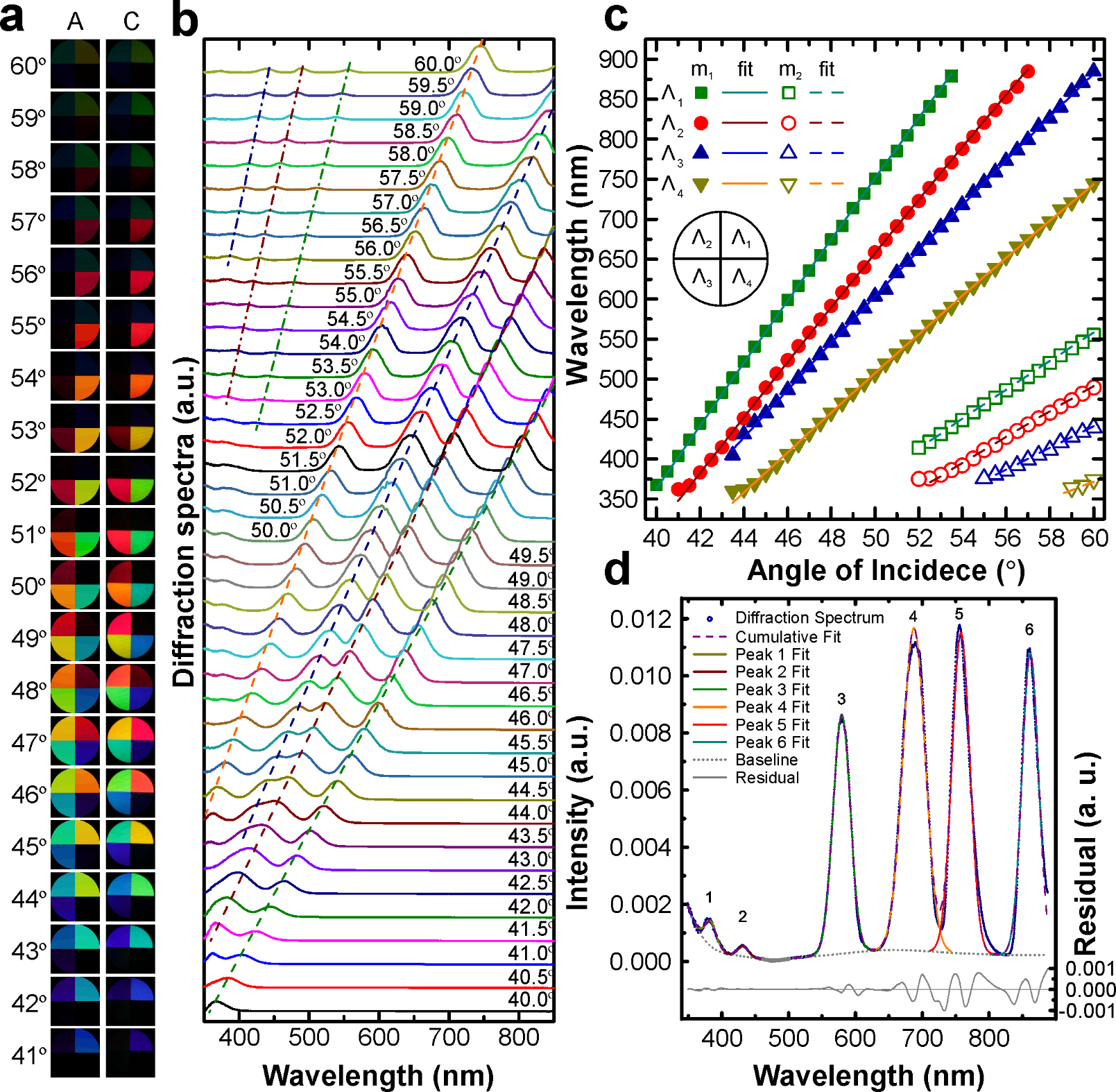


**Fig. S1** Extended test hologram experiment. Rendered hologram images (a, left column) next to actual camera snapshots (a, right column) and spectral composition (b) of the diffraction spectra obtained from a test hologram with four different grating periods (Λ_1_=1.328 µm, Λ_2_=1.175 µm, Λ_3_=1.016 µm, Λ_4_=0.8625 µm, see Fig. 8 (c)) acquired under different angles of incidence. Truncated and truncated dotted lines in (b) are guiding the readers eye along the peaks representing first and second diffraction orders respectively. Angle values indicated on the spectra and next to the images depict values of the corresponding angles of incidence θ_i_. (c) Summarizes the spectral peak position dependence on angle of incidence of the four different pitch holograms. The lines are fits using the diffraction grating equation (eq. S1). Solid lines depict the first diffraction order (*m*_1_) and truncated lines represent the second order (*m*_2_). (d) Example of the experimental diffraction spectra analysis of the four different pitch test hologram acquired at 53° angle of incidence. The experimental data were fitted employing 6 Normal distribution functions and background subtraction. Solid grey curve depicts residual of the fit.


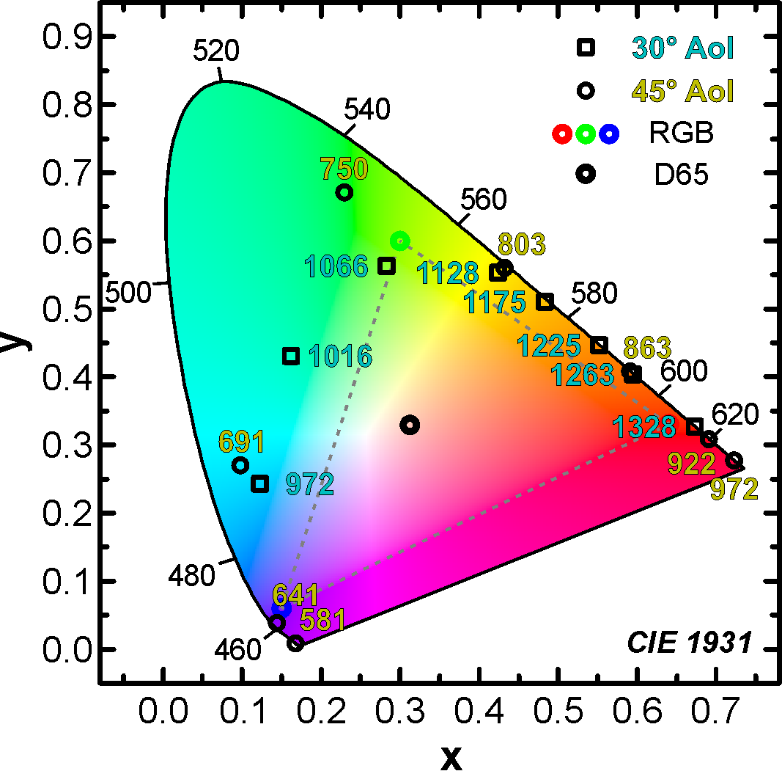


**Fig. S2** Colors that can be reconstructed from 15 different grating pitches, illuminated at 30° and 45° angle of incidence and observed at 0° (normal to the surface), available in the used DLIP setup.

| 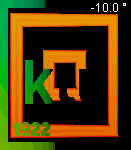 | 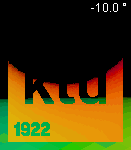 | 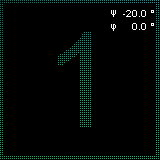 |
| --- | --- | --- |
| **Fig. S3** Rendered diffraction images at identified Ψ angles of the university logo demonstrating zooming squares effect (accompanied by a corresponding GIF file) | **Fig. S4** Rendered diffraction images at identified Ψ angles of the university logo demonstrating zooming sphere effect (accompanied by a corresponding GIF file) | **Fig. S5** Rendered diffraction image of countdown series at identified Ψ and ϕ angles demonstrating orientation and pitch-based multiplexing (accompanied by a corresponding GIF file) |


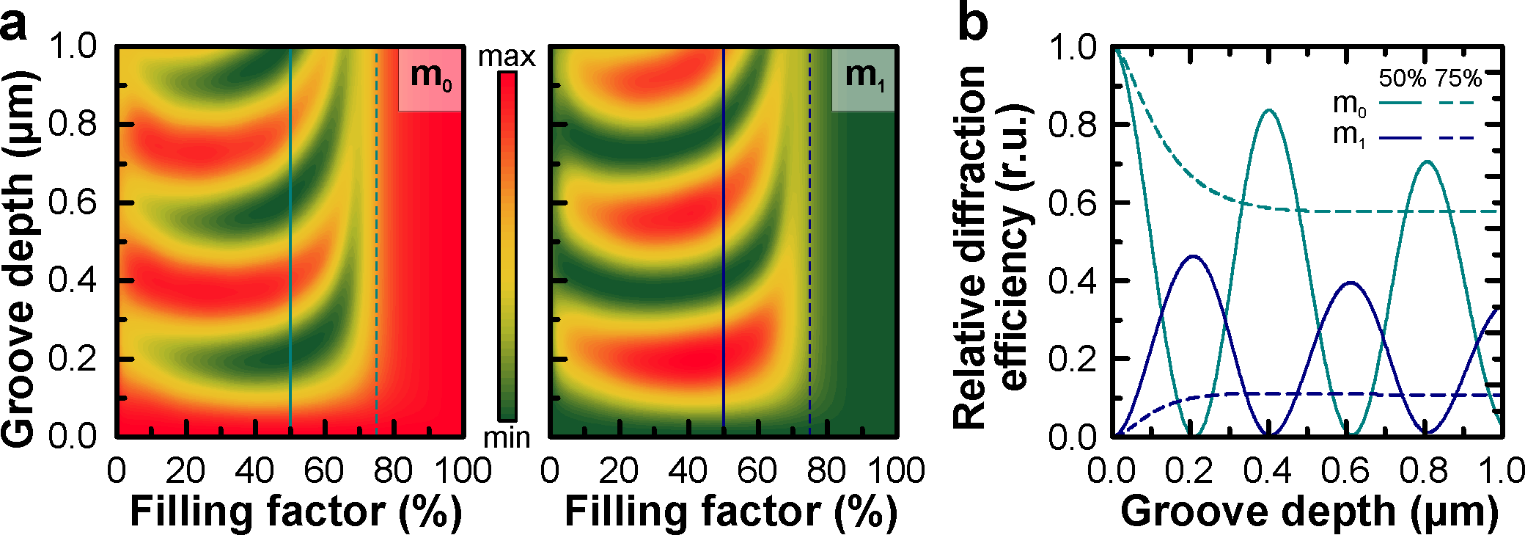


**Fig. S6** Diffraction efficiency simulation. Zero (*m*_0_) and first (*m*_1_) diffraction orders (a) were modeled for a perpendicular, TE polarized, 650 nm wavelength light incident on a structured nickel surface (periodicity 1016 nm). Carried out by changing the groove depth of the structure from 0 to 1000 nm with a step of 1 nm and varying the structure fill factor from 0 to 100% with a step of 1%. Solid and truncated vertical lines in (a) represent two characteristic filling factors (*f* = 50%, *f* = 75%). Diffraction efficiency dependence on groove depth for the filling factors is depicted in (b).


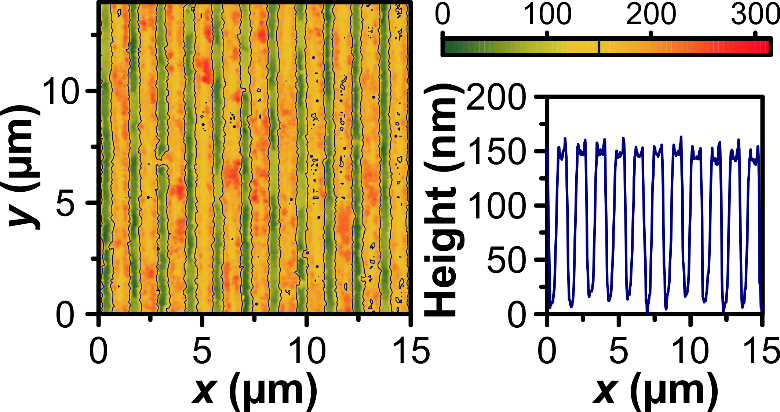


**Fig. S7** AFM micrograph of a diffraction grating produced at optimized processing conditions. Line graph shows a minimum depth profile of the grooves reaching 150 nm depth, which is also marked as a contour on the 2D image.


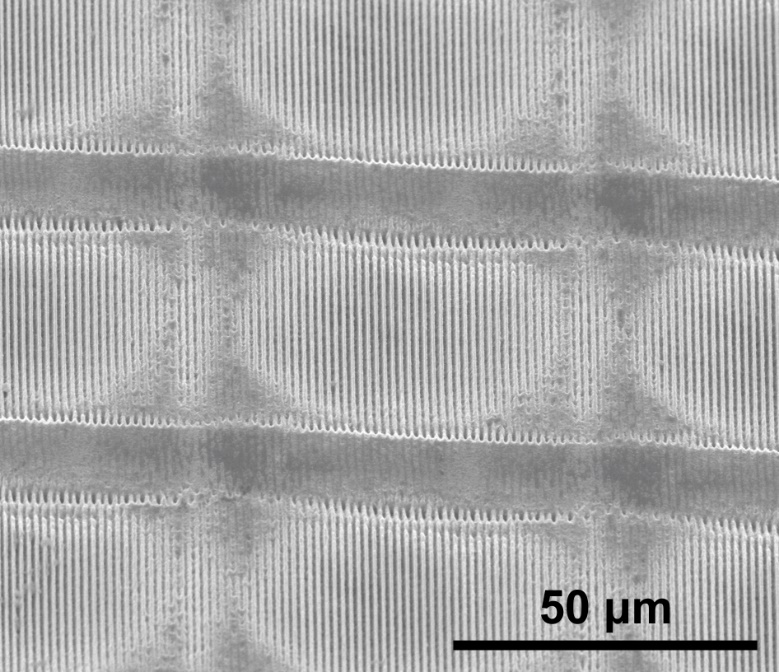


**Fig. S8** SEM micrograph of a 45° inclined test hologram surface ablated at 100100 pulses per pixel with fluence of 53.3 mJ/cm^2^.

**References**

1 Harvey, J. E. & Vernold, C. L. Description of diffraction grating behavior in direction cosine space. *Applied Optics* **37**, 8158-8160, doi:10.1364/ao.37.008158 (1998).

2 Allured, R. & McEntaffer, R. T. Analytical alignment tolerances for off-plane reflection grating spectroscopy. *Experimental Astronomy* **36**, 661-677, doi:10.1007/s10686-013-9349-y (2013).

3 Braig, C. *et al.* An EUV beamsplitter based on conical grazing incidence diffraction. *Optics Express* **20**, 1825-1838, doi:10.1364/oe.20.001825 (2012).

4 Jarasiunas, K. *et al.* Implementation of diffractive optical element in four-wave mixing scheme for ex situ characterization of hydride vapor phase epitaxy-grown GaN layers. *Review of Scientific Instruments* **78**, doi:10.1063/1.2712788 (2007).

5 Tamulevicius, T., Grazuleviciute, I., Jurkeviciute, A. & Tamulevicius, S. The calculation, fabrication and verification of diffraction grating based on laser beam splitters employing a white light scatterometry technique. *Optics and Lasers in Engineering* **51**, 1185-1191, doi:10.1016/j.optlaseng.2013.04.001 (2013).

6 Yaremchuk, I. *et al.* Numerical implementation of the S-matrix algorithm for modeling of relief diffraction gratings. *Journal of Modern Optics* **60**, 1781-1788, doi:10.1080/09500340.2013.861032 (2013).

7 Kondo, T., Juodkazis, S., Mizeikis, V., Misawa, H. & Matsuo, S. Holographic lithography of periodic two- and three-dimensional microstructures in photoresist SU-8. *Optics Express* **14**, 7943-7953, doi:10.1364/oe.14.007943 (2006).

8 Stankevicius, E., Gedvilas, M., Voisiat, B., Malinauskas, M. & Raciukaitis, G. Fabrication of periodic micro-structures by holographic lithography. *Lithuanian Journal of Physics* **53**, 227-237 (2013).

9 Jurkeviciute, A. *et al.* Fabrication and characterization of one- and two-dimensional regular patterns produced employing multiple exposure holographic lithography. *Journal of Optoelectronics and Advanced Materials* **19**, 119-126 (2017).
