# Supplementary figures and images for: Dot-Matrix Hologram Rendering Algorithm and its Validation through Direct Laser Interference Patterning
Source: Sci Rep. 2018 Sep 24;8:14245. doi: 10.1038/s41598-018-32294-5 (PMC6155248; doi:10.1038/s41598-018-32294-5)

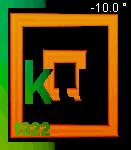

Supplement: Supplementary file 2 — Supplementary Figure S3 [file 41598_2018_32294_MOESM2_ESM.gif]

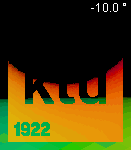

Supplement: Supplementary file 3 — Supplementary Figure S4 [file 41598_2018_32294_MOESM3_ESM.gif]

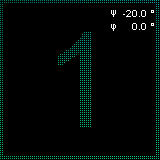

Supplement: Supplementary file 4 — Supplementary Figure S5 [file 41598_2018_32294_MOESM4_ESM.gif]
